# Older adults at‐risk for type 2 diabetes exhibit decreased performance on spatial and working memory tasks using classic and novel cognitive testing

**DOI:** 10.1002/alz.092717

**Published:** 2025-01-03

**Authors:** Olivia R Ghosh‐Swaby, Jennifer Hanna Al‐Shaikh, Daniel Palmer, Ali R. Khan, Jane Thornton, Timothy J Bussey, Lisa M Saksida, Teresa Liu‐Ambrose, Lindsay Nagamatsu

**Affiliations:** ^1^ Western University, London, ON Canada; ^2^ Rodent Cognition Research and Innovation Core, London, ON Canada; ^3^ Fowler Kennedy Sport Medicine Clinic, Western University, London, ON Canada; ^4^ University of British Columbia, Vancouver, BC Canada

## Abstract

**Background:**

Type 2 diabetes (T2D) and older age are well‐known risk factors for dementia. Indeed, there is evidence that older adults not diagnosed, but at‐risk for T2D can show early signs of cognitive decline, further exacerbated by excessive body weight or high blood glucose levels. Such a finding would have implications for early treatment strategies; however, the evidence is still sparse. We examined the correlation of risk factors for diabetes with cognitive function in older adults at‐risk for T2D using a battery of touchscreen tasks translated from their rodent versions, as well as traditional pen‐to‐paper cognitive tests.

**Method:**

Sixty‐five older adults (69.39 ± 10.31 years old, 68% female) at‐risk for T2D (BMI ≥ 25 kg/m2, hemoglobin A1c ≥ 6.0%, CANRISK score ≥ 21) completed 3 novel touchscreen tasks: paired associative learning (PAL) (learning and object‐in‐location memory), progressive ratio (motivation), and trial unique, non‐matching to location (TUNL) (spatial pattern separation and working memory). They were also tested on pen‐to‐paper cognitive tests: trail‐making (task switching), Stroop (selective inhibition), and digit span (working memory). A correlation analysis was performed between BMI or HbA1c and cognitive performance. Performance on touchscreen tasks was analyzed using a repeated measures one‐way ANOVA.

**Result:**

Higher HbA1c levels were correlated with lower digit span scores (r2 = 0.12, p = 0.011). There was no correlation between breakpoint (motivation level) and BMI or HbA1c during the progressive ratio task (r2 = 0.0, p = 0.38). Interestingly, this population performed at chance level (59.7 ± 5.3% accuracy) on the PAL task, indicating they were unable to learn object‐location paired associates. When manipulating the spatial similarity in distance between stimuli during the TUNL task, older adults at risk for diabetes were 10% lower in accuracy when stimuli were close together compared to further apart (p<0001). Participants also responded more slowly to stimuli at choice during the TUNL task during the heaviest working memory load condition (p = 0.003).

**Conclusion:**

Older adults at‐risk for T2D exhibit decreased performance on tasks with higher demands on spatial and working memory. Future research will compare performance on all tasks to healthy age‐matched controls and reassess performance after a six‐month exercise intervention.

